# Lightweight Siamese Network with Global Correlation for Single-Object Tracking

**DOI:** 10.3390/s24248171

**Published:** 2024-12-21

**Authors:** Yuxuan Ding, Kehua Miao

**Affiliations:** Department of Automation, Xiamen University, Xiamen 361102, China; 23220221151697@stu.xmu.edu.cn

**Keywords:** Siamese network, object tracking, cross-attention, lightweight

## Abstract

Recent advancements in the field of object tracking have been notably influenced by Siamese-based trackers, which have demonstrated considerable progress in their performance and application. Researchers frequently emphasize the precision of trackers, yet they tend to neglect the associated complexity. This oversight can restrict real-time performance, rendering these trackers inadequate for specific applications. This study presents a novel lightweight Siamese network tracker, termed SiamGCN, which incorporates global feature fusion alongside a lightweight network architecture to improve tracking performance on devices with limited resources. MobileNet-V3 was chosen as the backbone network for feature extraction, with modifications made to the stride of its final layer to enhance extraction efficiency. A global correlation module, which was founded on the Transformer architecture, was developed utilizing a multi-head cross-attention mechanism. This design enhances the integration of template and search region features, thereby facilitating more precise and resilient tracking capabilities. The model underwent evaluation across four prominent tracking benchmarks: VOT2018, VOT2019, LaSOT, and TrackingNet. The results indicate that SiamGCN achieves high tracking performance while simultaneously decreasing the number of parameters and computational costs. This results in significant benefits regarding processing speed and resource utilization.

## 1. Introduction

Object tracking represents a critical and complex challenge within the field of computer vision, with a diverse array of applications, including human–computer interaction, intelligent surveillance, autonomous driving, and medical diagnostics. This study involved the extraction of target information from the initial frame of a video, followed by continuous tracking of the target in the subsequent frames. Despite notable advancements in recent years, visual tracking continues to encounter numerous challenges, especially in the precise representation of targets during motion, encompassing issues related to deformation, rotation, and background occlusion. In practical applications, models are frequently implemented on edge devices that have hardware and power constraints, thereby complicating the deployment process.

**Figure 1:**
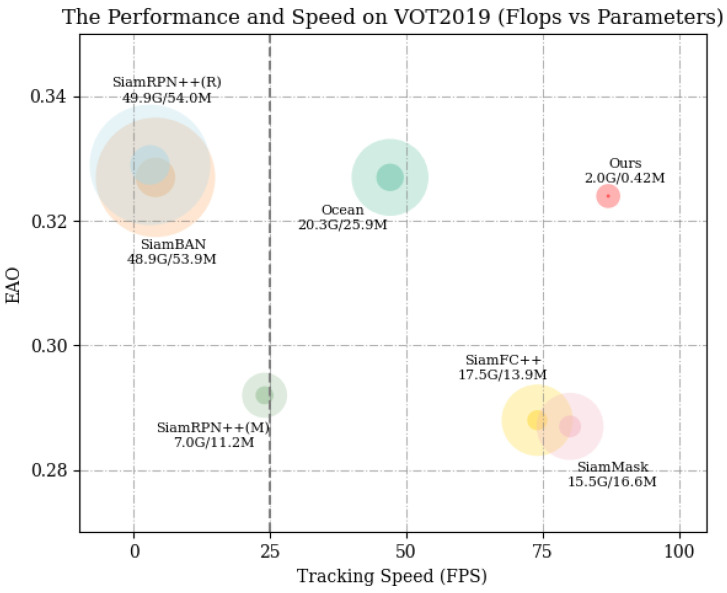
Comparison of state-of-the-art trackers according to the expected average overlap (EAO) and tracking speed (FPS) on VOT2019. Larger circles represent FLOPs, while smaller circles indicate parameters. A higher EAO and FPS are preferable, indicating better performance and faster tracking. Our method achieves a good balance between performance and efficiency, with a competitive EAO and high FPS, while maintaining low FLOPs and parameters.

In recent years, advancements in deep neural networks have led to substantial progress in the field of object tracking, especially with the development of Siamese-based trackers. Nonetheless, practical implementations generally require trackers to attain real-time or superior performance on edge devices. However, for the majority of current trackers, tracking speed is sacrificed in order to optimize their performance in benchmark evaluations. The efficacy of these trackers is contingent upon the utilization of advanced GPUs to facilitate real-time processing, resulting in limitations that are deemed unacceptable in practical applications. For instance, the groundbreaking SiamFC [1] requires 2.7 G FLOPs and comprises 2.3 M parameters. Furthermore, the more recent SiamBAN [2] and Ocean [3] trackers demand 48.9 G and 20.3 G FLOPs, with parameter counts of 54.0 M and 25.9 M, respectively (refer to Figure 1). This study presents the design of a more lightweight network achieved through modifications to MobileNetV3-small [4], which is part of the MobileNet series, to function as the backbone for feature extraction.

Recently, the Transformer [5] architecture has been successfully applied across various vision tasks [6,7,8]. In the domain of object tracking, the Transformer architecture has been integrated due to its exceptional abilities in global and dynamic modeling, facilitating attention-based feature fusion, as evidenced by various implementations such as TransT [9], TMT [10], STMTrack [11], MixFormer [12], STARK [13], and DTT [14]. Nonetheless, the Transformer architecture exhibits a considerable reliance on serial computation, resulting in a computational load that escalates in relation to the square of the number of input tokens. As a result, the computational complexity inherent in standard Transformers restricts these trackers from fully utilizing its architectural modeling capabilities. This limitation is particularly pronounced due to the significant computational expense linked to matrix multiplication during attention calculations. Mehta et al. [15] tackled this challenge by replacing costly matrix multiplications with separable elementwise operations, resulting in the creation of an efficient mobile vision Transformer (Mobile-ViT) module tailored for vision tasks. This study presents a novel approach to global feature fusion for real-time tracking, drawing inspiration from the fundamental principles of Transformer architectures and attention mechanisms to enhance efficiency.

This paper presents several key contributions:

- This study proposed a feature fusion module that leverages the attention mechanism in conjunction with vision Transformers (ViT) [7], which efficiently integrate features from the template and search region, enhancing representation without introducing additional latency.
- Our work developed an innovative lightweight tracking architecture, which achieves an AUC of 52.6% on LaSOT [16], utilizing only 2.4% of the parameters and 14.4% of the FLOPs compared to state-of-the-art methods, and maintaining a processing speed of 87 FPS on CPUs.
- Comparative analyses validate the effectiveness of the global correlation module and its integration with lightweight convolutional neural networks, showcasing superior performance on tracking benchmarks.

## 2. Related Work

### 2.1. Siamese Network-Based Trackers

Object tracking using Siamese networks has gained significant traction in recent years. The methods employed facilitate tracking through the development of a matching function that quantifies feature similarity between the template and the search region. SiamFC [1] represents a seminal contribution to this area of research. The approach utilizes a fully convolutional Siamese network that includes feature extraction and cross-correlation layers. This architecture facilitates the assessment of similarity between the template in the initial frame and the search region in later frames. The similarity is computed by employing template features as convolutional kernels, followed by executing a convolution operation within the search region. SiamRPN [17] advanced this concept by combining the Siamese network with a region proposal network (RPN) [18]. The system incorporated an up-channel cross-correlation layer that sequentially combined several independent cross-correlation layers to generate multi-channel response maps, and used RPN as the prediction head. The implementation of this design resulted in a notable enhancement in the precision of tracking capabilities. Ocean [3] introduced an anchor-free object-tracking network that directly forecasts the position and dimensions of the target. Through the implementation of comprehensive training for each pixel contained within the predicted bounding box, the model attains improved localization accuracy and overall precision. The tracker that operates without anchors, SiamBAN [2], simplifies the intricacies associated with anchor hyperparameter tuning by employing a unified fully convolutional network that concurrently classifies objects and regresses their bounding boxes at every spatial location. Nevertheless, the sophistication of these advanced trackers has escalated, necessitating a greater number of parameters and enhanced computational resources. This paper introduces a novel lightweight anchor-free network architecture, drawing inspiration from SiamBAN, with the objective of minimizing both parameters and FLOPs.

### 2.2. Feature Fusion Correlation

**Figure 2:**
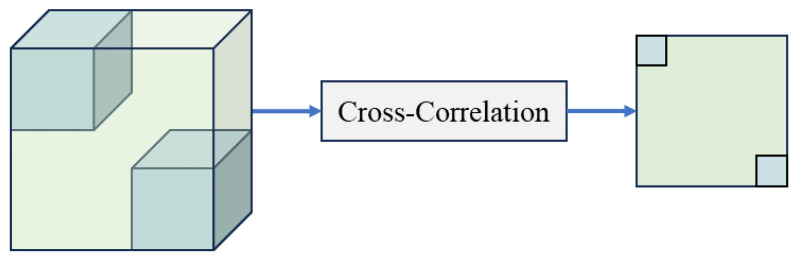
Cross-correlation for feature fusion. The blue squares represent the convolution kernels derived from the template features, while the green squares represent the feature maps obtained from the search region. Cross-correlation generates a single-channel correlation response map.

**Figure 3:**
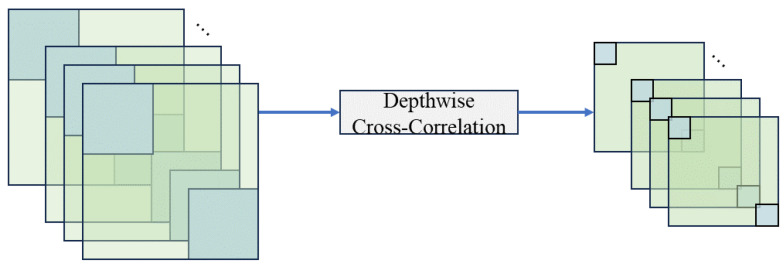
Depthwise cross-correlation for feature fusion. The blue squares and green squares represent the same as cross-correlation. Independent sliding kernels are applied to each channel of the template features and the search region features, producing a multi-channel correlation response map.

In numerous widely used trackers, including SiamFC [1], SiamRPN [17], and SiamRPN++ [19], feature fusion is generally executed through convolution-based correlation. This process is designed to linearly align the template or target data with the search region to evaluate their similarity. As demonstrated in Figure 2 and Figure 3, cross-correlation identifies the central area of the template features obtained during the feature extraction process. This central region is utilized as the convolution kernel, which is then applied to the search region, resulting in single-channel response maps. Conversely, depthwise cross-correlation emphasizes computational efficiency by performing correlation calculations independently for each feature channel, thereby producing multi-channel response maps. While this method effectively minimizes computational complexity, it may fail to account for interactions within the channel. The capacity to fully leverage global information in both methods is constrained by the inevitable loss of semantic information, which arises from the use of sliding windows and linear multiplication. The tracker may consequently become trapped in local optima, which restricts its capacity to comprehend intricate nonlinear interactions between the template and the search region.

Transformer [5], originally proposed by Vaswani et al., was first utilized in the domain of machine translation. The Transformer architecture fundamentally employs attention-based encoders and decoders to facilitate the conversion of one sequence into another. The model’s sensitivity to essential information is enhanced through the allocation of increased weight to the most pertinent input features. Recently, the application of attention mechanisms has been investigated within the domain of object tracking. SiamAttn [20] explores the utilization of self-attention and cross-branch attention mechanisms to improve the discriminative capacity of target features. The network is composed of two components: deformable self-attention, which emphasizes the comprehensive learning of contextual information, and cross-attention, which facilitates the interaction between the template and the search branch throughout the feature extraction process. In a similar vein, CGCAD [21] employs correlation-guided attention mechanisms, which markedly improve feature discrimination and bolster model robustness in the context of object tracking. TransT [9] features a Transformer-based fusion network that employs self-attention and cross-attention layers to enhance the integration of template and search region characteristics. The cross-attention mechanism facilitates the comprehensive nonlinear integration of input data, while its expansive receptive field diminishes the inductive bias typically associated with convolution-based correlation techniques. This methodology significantly enhances the quality of feature representation and improves the accuracy of tracking. Drawing upon the findings from these studies, a cross-attention-based feature fusion network, named global correlation, was designed. This approach effectively captures the intricate relationships between the template and search area while simultaneously preserving low computational complexity, thereby facilitating enhanced accuracy and real-time tracking capabilities.

## 3. Proposed Method

**Figure 4:**
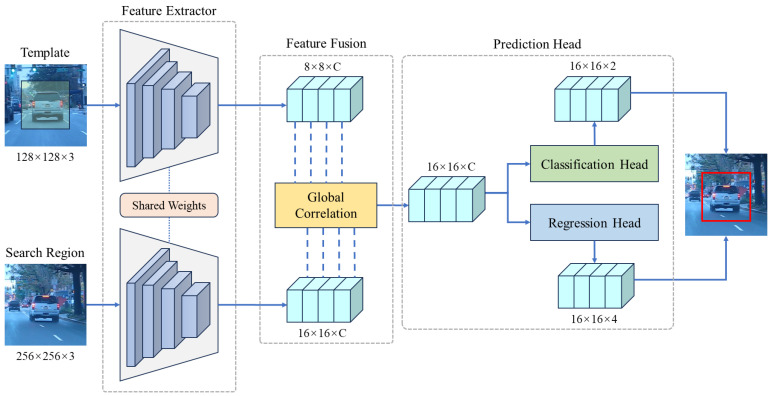
The SiamGCN architecture comprises three primary components: a feature extractor, a feature fusion network, and a prediction head. The template and search regions undergo processing via shared weights within the feature extractor. The extracted features are subsequently integrated through the global correlation module. The prediction head comprises distinct branches for both classification and regression tasks. The classification branch is responsible for predicting the correspondence of each region to either the foreground or background, whereas the regression branch is tasked with predicting the bounding box dimensions.

This section presents a detailed description of the proposed SiamGCN network, as depicted in Figure 4. SiamGCN is composed of three integral components: a feature extraction module, a feature fusion module, and a bounding box prediction network. The feature extraction module plays a crucial role in deriving features from both the search and template branches. The feature fusion module subsequently integrates these features through lightweight global correlation, which efficiently combines feature information from both branches. This approach simultaneously decreases the number of parameters while enhancing tracking accuracy. Subsequent to the process of feature fusion, the prediction head is utilized on the integrated feature vectors to execute bounding box regression and binary classification tasks. The selection of the bounding box is ultimately determined by the confidence score. This section provides a comprehensive analysis of each component of the SiamGCN, focusing on its lightweight architecture and the design of the global feature fusion module.

### 3.1. Siamese Network Architecture

**Feature Extraction.** To effectively deploy trackers on mobile platforms, it is essential that the feature extraction network exhibits flexibility, lightweight characteristics, and high accuracy. Moreover, the backbone network is required to produce features that possess a high spatial resolution, facilitating accurate target localization, while simultaneously minimizing the computational burden on subsequent layers. In alignment with the majority of Siamese networks [2,17,22,23], our model uses the template patch ($Z\in R^{3\times H_{z0}\times W_{z0}}$) and the search region patch ($X\in R^{3\times H_{x0}\times W_{x0}}$) as the inputs of SiamGCN. Here, $H_{z0}$ and $W_{z0}$ denote the height and width of the template patch extracted from the original input image, while $H_{x0}$ and $W_{x0}$ represent the height and width of the search region patch from the original input image. These dimensions are predefined during the data preprocessing stage to ensure consistency and compatibility with the feature extraction and correlation mechanisms of the model. The template patch is generated by expanding the bounding box of the initial target by a factor of two, whereas the bounding box of the target from the preceding frame is expanded by a factor of four. The patches are subsequently resized into square shapes prior to being input into the backbone network for the purpose of feature extraction. A modified variant of MobileNetV3-small [4] is utilized as the backbone network architecture. In particular, the stride of the final layer is decreased from 32 to 16. Compared to a stride of 32, reducing the stride to 16 improves the spatial resolution of the feature map, which is crucial for accurate target localization. While a stride of 32 results in extremely coarse feature maps that lack sufficient spatial detail, a stride of 16 offers a better balance by preserving more spatial information. This adjustment ensures that the backbone produces features with higher granularity, enabling more precise modeling of target boundaries and positions during the tracking process.

In contrast to earlier Siamese trackers [2,19], our study adjusted the backbone stride to 16 rather than 8. The spatial resolution of the output features derived from the backbone is consequently diminished, which results in a 75% reduction in computational expenses while maintaining efficacy in subsequent fusion and prediction processes. The enhancement in speed, while minimal on high-performance GPUs, is markedly substantial on edge devices, as illustrated in Section 4.3.

Finally, our work implemented a 1 × 1 convolution layer to modify the channel dimensions of the backbone features and subsequently flatten them in a spatial manner. This procedure generates two distinct sets of feature vectors, a template feature vector $f_{z}\in R^{C\times H_{z}\times W_{z}}$ and a search feature vector $f_{x}\in R^{C\times H_{x}\times W_{x}}$, where $H_{z},W_{z}=\frac{H_{z0}}{16},\frac{W_{z0}}{16}$ and $H_{x},W_{x}=\frac{H_{x0}}{16},\frac{W_{x0}}{16}$, with C=128. This adjustment ensures compact yet informative outputs, contributing to the overall efficiency and performance of the SiamGCN tracker.

**Feature Fusion.** The present work designed a feature fusion network that effectively integrates the template features $f_{z}$ and the search features $f_{x}$. As shown in Figure 4, the feature fusion network takes $f_{z}\in R^{C\times H_{z}\times W_{z}}$ and $f_{x}\in R^{C\times H_{x}\times W_{x}}$ as inputs from the template and search branches, respectively. These features are then fused through the global correlation module, which leverages an attention mechanism to produce the fused vector $f_{c}\in R^{d\times H_{c}\times W_{c}}$, where $H_{c},W_{c}=\frac{H_{x0}}{16},\frac{W_{x0}}{16}$. This process effectively harnesses the strengths of attention mechanisms, focusing on global feature relationships rather than local patterns emphasized in traditional convolution-based fusion methods, thereby optimizing feature integration in the tracking model. Based on our results, this method significantly improves tracking accuracy and robustness under various challenging scenarios. Detailed descriptions of the global correlation module are provided in Section 3.2.

**Prediction Head Network.** The prediction head is composed of two distinct branches: one dedicated to classification and another to regression. The classification branch determines the likelihood that each point on the response map is associated with either the foreground or the background. In contrast, the regression branch calculates the displacement between each point aligned with the search area and the actual ground truth bounding box. The ultimate prediction results are presented as follows:

(1) $$\begin{array}{cc} P_{W_{p}\times H_{p}\times 2}^{\mathrm{cls}} & =H_{\mathrm{cls}}\left(f_{c}\right),\\ P_{W_{p}\times H_{p}\times 4}^{\mathrm{reg}} & =H_{\mathrm{reg}}\left(f_{c}\right), \end{array}$$

where $H_{p},W_{p}=\frac{H_{x0}}{16},\frac{W_{x0}}{16}$. $H_{\mathrm{cls}}\left(\cdot \right)$ and $H_{\mathrm{reg}}\left(\cdot \right)$ represent the classification head and the bounding box regression head, respectively. In the context of classification and regression outputs *P*, $P_{i,j}^{\mathrm{cls}}=\left(p_{f},p_{b}\right)$ represents the foreground and background at a specific location within the test image, while $P_{i,j}^{\mathrm{reg}}=\left(l,t,r,b\right)$ indicates the distances from that location to the four edges of the bounding box in the test image.

**Figure 5:**
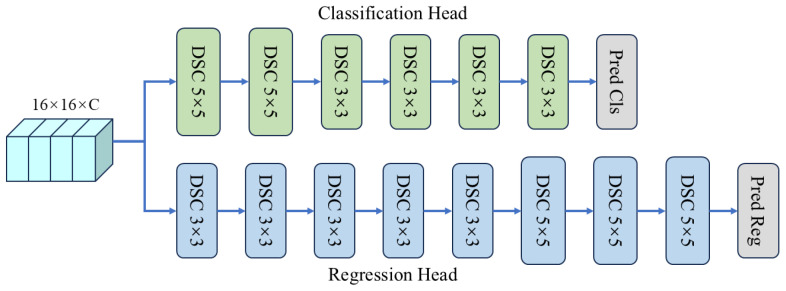
Prediction head architecture. Both heads are composed of several depthwise separable convolution (DSC) layers.

Figure 5 illustrates that our tracker employs several depthwise separable convolution (DSConv) blocks. When contrasted with conventional convolution operations, these blocks exhibit a reduction in the number of parameters and a decrease in computational expenses. The regression task necessitates accurate predictions of bounding box coordinates, thereby requiring a comprehensive analysis to effectively capture spatial relationships and nuanced variations in object size. Consequently, an increased number of DSConv blocks is employed within the regression head to accurately estimate the distances between points on the feature map and the corresponding edges of the original image. Figure 5 illustrates that the classification head incorporates a reduced number of DSConv blocks in comparison to the regression head. This design facilitates the efficient sharing of learned feature representations between the classification head and the bounding box regression head. The simplified network architecture concurrently diminishes the number of parameters and computational demands, thereby enhancing its suitability for implementation on edge devices.

### 3.2. Global Correlation

**Figure 6:**
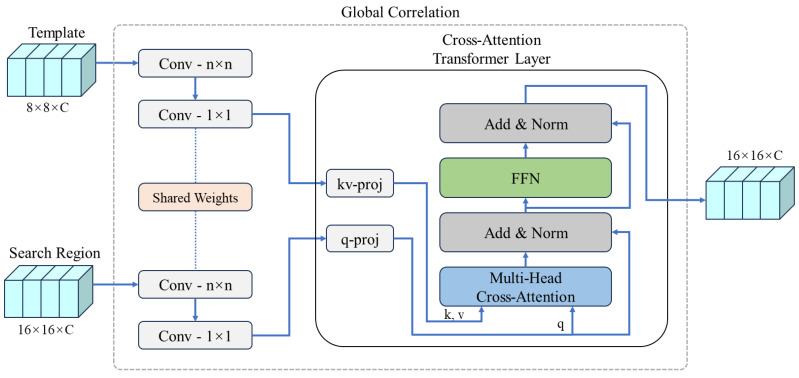
The global correlation module leverages a cross-attention Transformer layer, designed based on multi-head attention, to fuse features from the template and search branches. Queries *Q* are generated from the search branch, while keys *K* and values *V* are derived from the template branch. The multi-head cross-attention mechanism captures global relationships between features, followed by normalization and a feed-forward network (FFN) for enhanced feature stability.

The architecture of the global correlation network is shown in Figure 6. The inputs are two feature maps with dimensions of 8×8×C and 16×16×C, both processed using convolutional layers with shared weights. Each set of features first passes through a standard n×n convolutional layer to encode local spatial information. Then, the features are projected into a higher-dimensional space (or *d*-dimensional space, where d=144 and C=96) via pointwise convolutions (also known as 1×1 convolutions). In addition to reducing the number of parameters, the model ensures consistency and efficiency when processing features of different sizes by convolution with shared weights.

The cross-attention Transformer layer is designed based on multi-head attention. Given *Q*, *K*, and *V*, representing the query (from the search branch), key, and value (from the template branch), respectively, the cross-attention function is defined as

(2) $$\mathrm{Cross}-\mathrm{Attention}\left(Q,K,V\right)=\mathrm{softmax}\left(\frac{QK^{T}}{\sqrt{d}}\right)V.$$

As proposed by Vaswani et al. [5], to capture information at different levels, the attention mechanism can be extended to multi-head attention, allowing the model to consider diverse attention distributions. Multi-head cross-attention is defined as

(3) $$\mathrm{MultiHead}\left(Q,K,V\right)=\mathrm{Concat}\left(H_{1},H_{2},\cdots ,H_{n_{h}}\right)W^{O}.$$

where $H_{i}$ is the attention output of the *i*-th head, defined as

(4) $$H_{i}=\mathrm{Cross}-\mathrm{Attention}\left(QW_{i}^{Q},KW_{i}^{K},VW_{i}^{V}\right).$$

where $W_{Q_{i}}\in R^{d_{m}\times d_{k}},$$W_{K_{i}}\in R^{d_{m}\times d_{k}},$$W_{V_{i}}\in R^{d_{m}\times d_{v}},$ and $W_{O}\in R^{n_{h}d_{v}\times d_{m}}$ are parameter matrices. In this study, we use $n_{h}=4$, $d_{m}=144$, and $d_{k}=d_{v}=\frac{d_{m}}{n_{h}}=36$ as default values.

In the cross-attention Transformer layer, the query *Q* for the search branch is generated using a linear projection layer (q-proj), while the key *K* and value *V* for the template branch are generated using another linear projection layer (kv-proj). Then, they are passed into the multi-head cross-attention, which computes the weighted sum of attention across different heads, effectively fusing the information from search and template branches. After the cross-attention layer, an Add & Norm operation is applied, followed by a feed-forward network (FFN). The FFN consists of two linear layers with a ReLU activation function to introduce nonlinearity. Another Add & Norm operation is then performed to ensure feature stability. Finally, a 1 × 1 convolution layer is applied, producing a 16 × 16 × C similarity response map, which is used as the input for the subsequent prediction head network.

The proposed global correlation effectively leverages the strengths of the attention mechanism, focusing more on global feature relationships than local features emphasized in traditional convolution-based fusion methods. By capturing long-range dependencies between the template and search region, the module robustly models global contextual relationships, which are essential for addressing challenging tracking scenarios such as occlusions and cluttered backgrounds. The integration of pointwise convolutions ensures consistent feature representations while maintaining computational efficiency by reducing parameter overheads. Furthermore, the multi-head attention mechanism enables parallel processing, balancing the computational cost with significant performance improvements. This cohesive design not only enhances the robustness of the tracker but also improves its adaptability to varying object sizes and motions, ultimately optimizing feature fusion and leading to superior tracking performance.

### 3.3. Training Loss

The model is trained utilizing a loss function that is applied to the outputs of both classification and regression produced by the prediction head. In the classification branch, cross-entropy loss is utilized, while the regression branch employs generalized intersection-over-union (IoU) loss [24]. The total loss function $L_{\mathrm{total}}$ is defined as

(5) $$L_{\mathrm{total}}=\lambda_{\mathrm{CE}}\cdot L_{\mathrm{CE}}+\lambda_{\mathrm{gIoU}}\cdot L_{\mathrm{gIoU}}.$$

where $L_{\mathrm{CE}}$ represents the cross-entropy loss, and $L_{\mathrm{gIoU}}$ represents the generalized IoU loss. Throughout the training process, it became evident that the performance of the classification branch considerably surpassed that of the regression branch. In order to achieve equilibrium in the training process, our work modified the weights assigned to the overall loss function corresponding to each branch. Specifically, we established the values of $\lambda_{\mathrm{CE}}=1$ and $\lambda_{\mathrm{gIoU}}=1.2$. This modification enhanced the training process of the regression branch, thereby improving the performance of the tracker in terms of localization accuracy.

## 4. Experiments

### 4.1. Implementation Details

**Training.** The entirety of our network is capable of being trained in an end-to-end manner utilizing extensive datasets. SiamGCN was trained utilizing pairs of images derived from both video sources and static images. The dataset utilized for training comprises ImageNet-VID [25], ImageNet-DET [25], GOT-10k [26], and COCO [27]. The size of the search region patch measures 256×256, while the template patch measures 128×128. In the context of the video datasets, specifically GOT-10k and ImageNet-VID, we employ a method of random selection whereby two frames are chosen from the same video sequence, ensuring that they are no more than 100 frames apart. This selection process is utilized to create the template and search region patches. Transformations are applied to the original images in the COCO and ImageNet-DET datasets to create sampled pairs. In the course of training, each epoch employs $6\times 10^{4}$ image pairs that are uniformly sampled from the training dataset.

The backbone parameters are initialized using MobileNetV3-small [4], which is pretrained on the ImageNet dataset [25]. The training process is divided into two distinct stages. In the initial phase, the backbone and head are subjected to training for a duration of 20 epochs. Initially, during the first five epochs, a warm-up learning rate is implemented, transitioning from 0.001 to 0.005. Subsequently, over the following fifteen epochs, the learning rate undergoes exponential decay, decreasing from 0.005 to 0.00005. The learning rate for the backbone is established at one-tenth of the prevailing learning rate. At this stage, the ADAM optimizer [28] with a weight decay of $10^{-4}$ is employed to facilitate rapid convergence of the model. During the subsequent phase, fine-tuning of the model is performed. The optimization process is transitioned to stochastic gradient descent (SGD), incorporating a weight decay parameter of 0.0001 and a momentum coefficient of 0.9. In the initial ten epochs of this phase, we maintain the backbone parameters in a fixed state, while ensuring that all other configurations remain consistent with those established in the preceding stage.

**Testing.** An offline tracking strategy is used throughout the testing phase. The object depicted in the initial frame of the video sequence serves as the template for subsequent analysis. Consequently, the target branch of the Siamese network can be pre-computed and kept constant during the tracking procedure. The search region derived from the present video frame serves as the input for the search branch. This study assessed various datasets by employing their designated official metrics, which can vary from one dataset to another. The tracker was developed using Python 3.7 and PyTorch 2.1.0. Training and testing processes were executed on a personal computer equipped with an Intel i7-12700H CPU and an Nvidia 3090Ti GPU. The training process for SiamGCN requires approximately 7 h, reflecting the computational efficiency of its lightweight architecture. SiamGCN is characterized by its simplicity as a tracker without post-processing, positional embedding, and multi-layer feature aggregation strategies.

### 4.2. Results and Comparisons

This research compares SiamGCN with state-of-the-art trackers on four tracking benchmarks, including VOT2018 [29], VOT2019 [30], LaSOT [16], and TrackingNet [31].

**VOT2018.** VOT2018 [29] consists of 60 challenging video sequences that encompass a range of scenarios, including rapid motion and occlusion. The overall performance of the tracker was assessed through the expected average overlap (EAO), which integrates two critical components: accuracy, defined as the average overlap achieved during successful tracking, and robustness, characterized by the failure rate. The results of the comparison with leading trackers on VOT2018 are illustrated in Table 1. Among the previously established methods, SiamBAN [2] attained the highest expected average overlap (EAO), whereas SiamMask [32] demonstrated the highest accuracy, with both methodologies utilizing ResNet50 for feature extraction. Our approach, characterized by its efficient design, attains the second-highest performance in terms of expected average overlap (EAO) and robustness. This is accomplished while markedly decreasing the number of parameters and FLOPs in comparison to the leading SiamBAN model. The tracker demonstrates elevated effectiveness and robustness, concurrently achieving a significant reduction in the consumption of computational resources.

**VOT2019.** The VOT2019 [30] dataset also consists of 60 challenging video sequences, with 20% of these sequences being updated from VOT2018. This update introduces further complexities, especially in scenarios involving rapid motion and similar distractors. The performance of our SiamGCN is illustrated in Table 2, showcasing metrics such as the expected average overlap (EAO), accuracy, and robustness. This comparison delineates SiamRPN++ (R) and (M), which correspond to the versions utilizing ResNet50 and the lightweight backbone MobileNetv2 for the purpose of feature extraction, respectively. In comparison to SiamRPN++ utilizing ResNet50, the implementation of MobileNetv2 led to a decrease of 11.2% in EAO, along with significant declines in accuracy and robustness. In contrast, our approach employs a lightweight backbone for feature extraction, attaining an EAO that is nearly equivalent to that of the second-best tracker, Ocean, while also exhibiting superior robustness. Furthermore, the parameters and FLOPs of SiamGCN constitute merely 2.1% and 7.7% of those of Ocean, respectively. SiamGCN effectively manages intricate scenarios, underscoring its applicability for implementation in environments with limited resources.

**LaSOT.** LaSOT [16] is a large-scale long-term tracking dataset that comprises 1120 training videos and 280 test videos. It encompasses 14 distinct attributes and maintains balanced categories. The typical duration of each video is 2500 frames. Table 3 illustrates that SiamGCN secures the top position in precision metrics while achieving second place in AUC and *P_Norm_*. Specifically, it attains 96% of the performance level of the state-of-the-art HiT model in AUC while requiring only 21% of HiT’s parameters and 42% of its FLOPs. This outcome illustrates the robust capability of SiamGCN in managing long video sequence tracking tasks, highlighting its commendable equilibrium between performance and efficiency.

**TrackingNet.** TrackingNet [31] is a large-scale dataset designed for the evaluation and development of object-tracking algorithms. The dataset comprises over 30,000 videos, which include more than 14 million meticulously annotated bounding boxes. The videos are predominantly obtained from YouTube and encompass a diverse array of real-world object categories and situations. The performance of SiamGCN was evaluated using its testing set. Table 4 illustrates that our SiamGCN exhibits competitive performance on TrackingNet, attaining a precision rate of 66.4%, surpassing SiamFC++ and ATOM by 1.8% and 1.6%, respectively. In terms of the AUC and normalized precision *P_Norm_*, SiamGCN achieves 71.5% and 76.8%, respectively, maintaining comparable results to HiT. Additionally, SiamGCN utilizes 79% fewer model parameters and 57% fewer FLOPs, highlighting its exceptional efficiency.

### 4.3. Ablation Studies

**Comparison of feature fusion.** This study evaluated the efficacy of the proposed global correlation method in comparison to two widely utilized correlation techniques within the context of the VOT2019 dataset. In native correlation, the central region of the template features $f_{z}\in R^{C\times H_{z}\times W_{z}}$, denoted as $f_{z}^{\mathrm{center}}\in R^{C\times \frac{H_{z}}{2}\times \frac{W_{z}}{2}}$, is selected as the convolution kernel and is utilized in a sliding window convolution across the search region features $f_{x}\in R^{C\times H_{x}\times W_{x}}$, producing a similarity response map. Here, *C* is the number of feature channels, while $H_{z},W_{z}$ and $H_{x},W_{x}$ represent the spatial dimensions of the template and search region, respectively, determined through preprocessing steps. Depthwise correlation exhibits a distinct approach by considering each channel of the template features $f_{z}^{\mathrm{center}}$ as an individual convolution kernel. It independently applies sliding window convolution to the corresponding channel of the search region features $f_{x}$, generating a similarity response map.

The computational complexities of native correlation, depthwise correlation, and the global correlation module differ significantly due to their mechanisms. Native correlation calculates the local similarity by treating the entire template feature $f_{z}$ as a convolutional kernel sliding over the search region $f_{x}$, with a complexity of $O(C^{2}\cdot H_{z}\cdot W_{z}\cdot H_{x}\cdot W_{x})$. Depthwise correlation, on the other hand, simplifies the process by treating each channel of the template feature as an independent kernel, performing sliding window convolutions independently for each channel. This reduces the computational complexity to $O(C\cdot H_{z}\cdot W_{z}\cdot H_{x}\cdot W_{x})$ but sacrifices the ability to capture inter-channel dependencies.

In contrast, the global correlation module leverages attention mechanisms to model global interactions between the template and the search region, with a complexity of $O(C\cdot \left(H_{z}\cdot W_{z}+H_{x}\cdot W_{x}\right)\cdot d+d\cdot H_{z}\cdot W_{z}\cdot H_{x}\cdot W_{x})$. The complexity is primarily determined by the second term $O(d\cdot H_{z}\cdot W_{z}\cdot H_{x}\cdot W_{x})$, which mainly depends on the projected feature dimension *d*. The global correlation module achieves significant improvements in robustness and tracking accuracy by effectively capturing rich global feature dependencies. Although this introduces a modest increase in computational complexity, the use of multi-head attention mechanisms enables parallel processing, which helps optimize computational performance and ensures the module remains efficient.

The results of the comparison involving our tracker and various correlation methods are illustrated in Table 5. In comparison to depthwise correlation and native correlation, our global correlation demonstrates enhancements in EAO of 5.5% and 7.2%, respectively, along with notable improvements in robustness. Furthermore, the global correlation module maintains a competitive computational efficiency, with FLOPs and parameter counts slightly higher than those of depthwise correlation but significantly lower than those of native correlation. This balance between superior tracking accuracy and computational cost demonstrates the module’s effectiveness and practicality for real-world applications. These findings indicate that global correlation exhibits superiority across multiple dimensions.

**Comparison of different backbones.** This study evaluated the performance of various backbone networks utilizing the VOT2019 dataset, as illustrated in Table 6. In order to optimize both efficiency and accuracy within the tracking model, the output feature stride of all the backbone networks was modified to 16. The evaluations were performed on a personal computer equipped with an Intel Core i7-12700 CPU and an NVIDIA GeForce RTX 3090 Ti GPU. FasterNet [39] exhibited remarkable performance on GPU platforms; however, its performance on CPU systems was comparatively subpar. This discrepancy suggests that the optimizations implemented in FasterNet are predominantly tailored for GPU utilization rather than CPU execution.

In comparison, MobileNetV3 [4] demonstrated stable and consistent performance across both GPU and CPU platforms. Designed specifically for mobile and edge devices, MobileNetV3 achieves a practical trade-off between accuracy and computational efficiency by incorporating depthwise separable convolutions, squeeze-and-excitation modules, and the optimized hard-swish activation function. Although FasterNet and MobileNetV3 demonstrated similar outcomes and exhibited strong performance on GPUs, the markedly inferior performance of FasterNet on CPUs makes it impractical for implementation on edge devices. Given these considerations, MobileNetV3 was selected as the backbone for the SiamGCN model to ensure high tracking accuracy and efficient operation on resource-constrained platforms.

**Comparison of loss function.** To assess the impact of different loss function configurations on tracking performance, we conducted experiments with varying weights for the classification and regression losses on the VOT2019 benchmark. The results are summarized in Table 7.

The configuration $\lambda_{\mathrm{CE}}=1,\lambda_{\mathrm{gIoU}}=1$ serves as the baseline, providing balanced performance and acting as a reference for further tuning. Increasing the regression weight to $\lambda_{\mathrm{gIoU}}=1.2$ improves localization accuracy and robustness, achieving the best overall performance. Conversely, emphasizing classification with $\lambda_{\mathrm{CE}}=1.2$ leads to a decline in performance, highlighting that excessive focus on classification compromises bounding box precision. Adjusting the weights to $\lambda_{\mathrm{CE}}=0.8,\lambda_{\mathrm{gIoU}}=1.2$ also yields competitive results, highlighting the importance of striking a balance between classification and regression contributions to achieve optimal tracking performance. These findings demonstrate that slightly increasing the weight of the regression branch can effectively improve the tracker’s performance.

### 4.4. Visualization of Tracking Results

**Figure 7:**
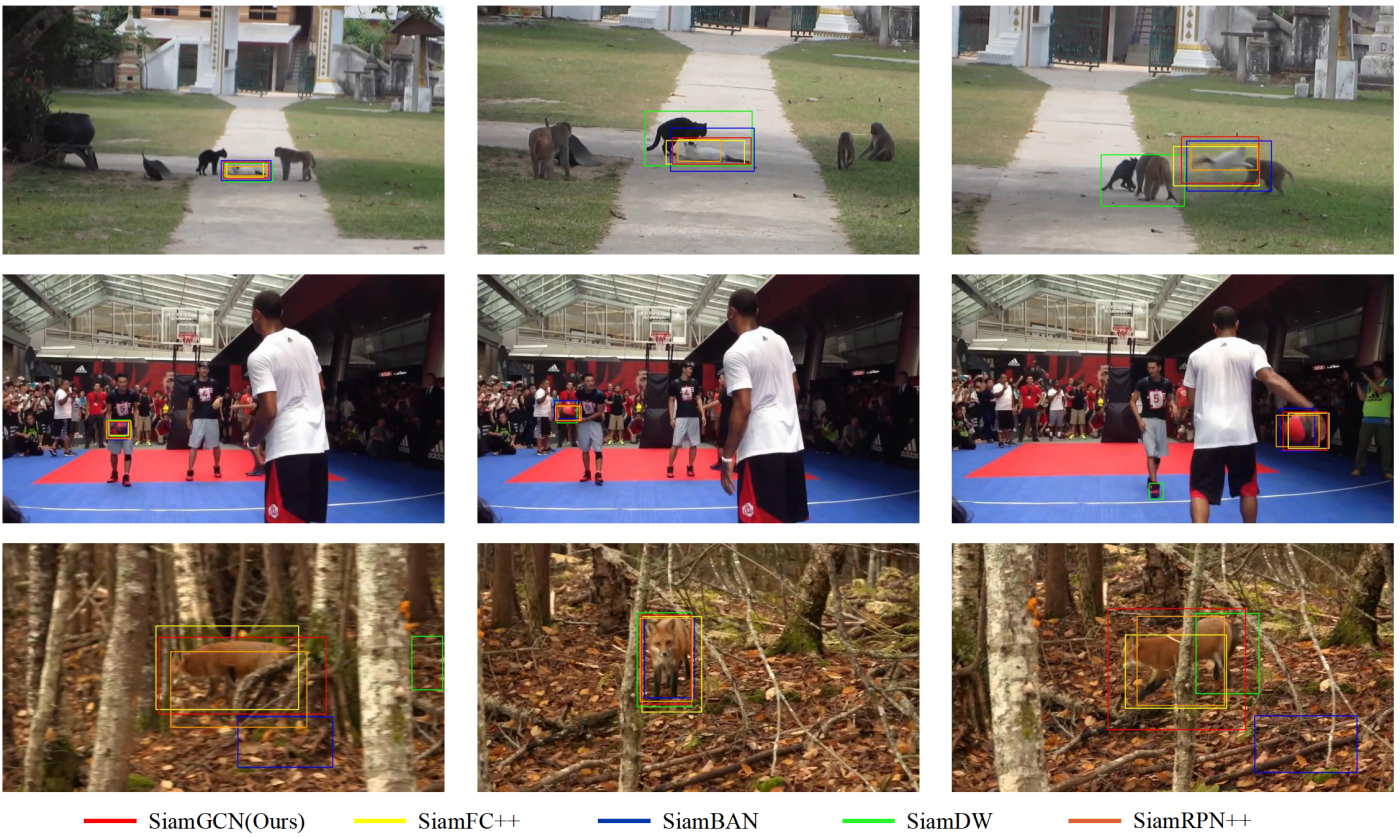
Visualizations of the tracking results of SiamGCN compared to other trackers, SiamFC++ [22], SiamBAN [2], SiamDW [35], and SiamRPN++ [19], across three representative video sequences from LaSOT [16]. The red bounding box corresponds to SiamGCN, which consistently demonstrates accurate tracking under challenging conditions, such as aspect ratio changes, fast motion, and partial occlusion. In contrast, the other trackers show coarser bounding boxes, often failing to tightly fit the target. These results indicate the superior tracking stability and accuracy of SiamGCN, especially in maintaining precise prediction even when the object undergoes significant changes or occlusion.

The visualization of tracking results is presented in Figure 7. The proposed model exhibits consistently stable tracking performance when evaluated across three representative video sequences. In comparison to the baseline model, our methodology demonstrates a marked enhancement in tracking accuracy. 

### 4.5. Limitations

**Figure 8:**
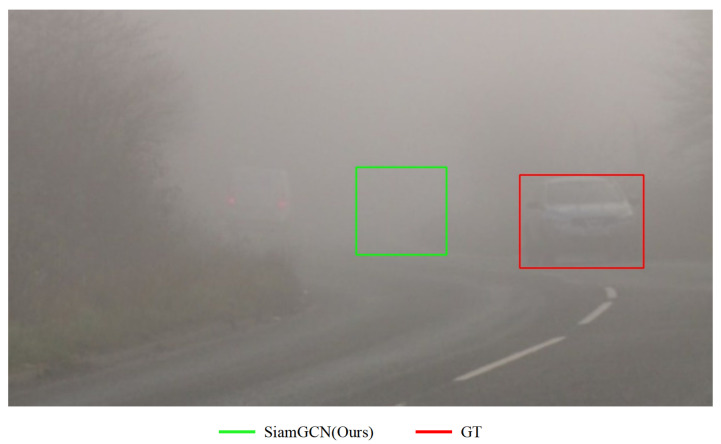
Visualization of the tracking results underscores the inherent challenges of object tracking under dense fog weather conditions. The red bounding box represents the ground truth, while the green bounding box denotes the tracking result produced by our model. The severely reduced visibility and indistinct features make it difficult for the model to consistently learn and capture feature information, highlighting the need for further optimization to enhance performance in such challenging scenarios.

While the proposed method demonstrates strong performance across various benchmarks and challenging scenarios, its effectiveness under adverse weather conditions, such as heavy rain, snow, or dense fog, remains limited. These environments introduce significant noise, occlusions, and distortions, which compromise the robustness of the tracking system. As illustrated in Figure 8, dense fog severely reduces visibility and obscures feature details, making it challenging for the model to consistently learn and capture target information. As highlighted in related studies [44,45], such conditions result in less distinguishable features and degraded tracking accuracy. To address these challenges, future work will explore integrating image enhancement and restoration techniques into the tracking pipeline.

## 5. Conclusions

This paper presents the design of a lightweight object tracker named SiamGCN, which is composed of three integral components: feature extraction, feature fusion, and a prediction head. Our work adapted MobileNet-V3 to meet the specifications of the tracking model, utilizing it as the backbone for feature extraction. SiamGCN incorporates a multi-head cross-attention fusion module, termed global correlation, for the purpose of feature fusion. In contrast to conventional sliding window convolution-based techniques, our methodology emphasizes the incorporation of global information. Extensive experiments were conducted utilizing four widely recognized benchmark datasets. The findings indicate that, despite SiamGCN exhibiting markedly reduced FLOPs and a lower number of parameters in comparison to the other widely used trackers, its performance on specific benchmarks did not attain the state-of-the-art standard. In future research, we intend to investigate advanced network architectures and integration methodologies to optimize the equilibrium between tracking efficacy and model efficiency.

## Figures and Tables

**Table 1:** Detailed comparisons on VOT2018. The best and second best results are highlighted in red and blue fonts, and the same rule applies to the tables below.

	ECO	SiamMask	SiamRPN	SiamRPN++	HiT	SiamBAN	SiamGCN
	[33]	[32]	[17]	[19]	[34]	[2]	
EAO (↑)	0.281	0.380	0.384	0.414	0.425	0.452	0.429
Accuracy (↑)	0.484	0.609	0.586	0.600	0.591	0.597	0.578
Robustness (↓)	0.276	0.276	0.276	0.234	0.213	0.178	0.197
FLOPs (G) (↓)	35.6	15.5	21.9	48.9	0.99	48.9	0.42
Parameters (M) (↓)	34	16.6	90.4	54.0	9.59	53.9	2.0

**Table 2:** Detailed comparisons on VOT2019.

	SiamMask	SiamFC++	SiamRPN++(M)	HiT	SiamRPN++(R)	Ocean	SiamGCN
	[32]	[22]	[19]	[34]	[19]	[3]	
EAO (↑)	0.287	0.288	0.292	0.312	0.329	0.327	0.324
Accuracy (↑)	0.594	0.583	0.580	0.605	0.590	0.602	0.589
Robustness (↓)	0.461	0.406	0.446	0.408	0.376	0.396	0.373
FLOPs (G) (↓)	15.5	17.5	7.0	0.99	48.9	20.3	0.42
Parameters (M) (↓)	16.6	13.9	11.2	9.6	54.0	25.9	2.0

**Table 3:** Detailed comparisons on LaSOT.

	ECO	SiamDW	SiamMask	SiamRPN++	SiamBAN	HiT	SiamGCN
	[33]	[35]	[32]	[19]	[2]	[34]	
AUC (%) (↑)	32.4	38.4	46.7	49.6	51.4	54.8	52.6
*P_Norm_* (%) (↑)	33.8	47.4	55.2	56.9	59.8	60.5	60.1
P (%) (↑)	30.1	38.1	46.9	49.1	52.1	52.9	53.0
FLOPs (G) (↓)	35.6	35.6	15.5	48.9	48.9	0.99	0.42
Parameters (M) (↓)	34	34	16.6	54.0	53.9	9.6	2.0

**Table 4:** Detailed comparisons on TrackingNet.

	ECO	DaSiam	C-RPN	ATOM	SiamFC++	HiT	SiamGCN
	[33]	[36]	[37]	[38]	[22]	[34]	
AUC (%) (↑)	55.4	63.8	66.9	70.3	71.2	74.6	71.5
*P_Norm_* (%) (↑)	61.8	73.3	74.6	77.1	75.8	78.1	76.8
P (%) (↑)	49.2	59.1	61.9	64.8	64.6	68.8	66.4
FLOPs (G) (↓)	35.6	21.0	—	—	17.5	0.99	0.42
Parameters (M) (↓)	34	19.6	—	8.4	13.9	9.6	2.0

**Table 5:** Comparison of different correlation methods on VOT2019.

Correlation Method	EAO (↑)	Accuracy (↑)	Robustness (↓)	Params (↓)	Flops (↓)
Native Correlation	0.302	0.575	0.436	4.34	1.14
Depthwise Correlation	0.307	0.581	0.447	1.94	0.37
Global Correlation	0.324	0.589	0.373	2.01	0.42

**Table 6:** Comparison of different backbones of SiamGCN.

Backbone	Params (M) (↓)	FLOPs (G) (↓)	EAO (↑)	Accuracy (↑)	Robustness(↓)	CPU (FPS)	GPU (FPS)
ResNet18 [40]	12.8	4.28	0.296	0.583	0.406	10	176
ResNet50 [40]	26.2	8.46	0.313	0.594	0.385	6	202
ShuffleNetV2 [41]	3.31	1.02	0.287	0.591	0.482	51	358
EfficientNet [42]	6.42	0.83	0.275	0.577	0.507	53	355
MobileNetV2 [43]	4.64	0.72	0.305	0.581	0.384	48	349
FasterNet [39]	3.35	2.06	0.321	0.592	0.390	23	753
MobileNetV3 [4]	2.01	0.42	0.324	0.589	0.373	87	492

**Table 7:** Comparison of different loss function configurations on VOT2019.

Configuration	EAO (↑)	Accuracy (↑)	Robustness (↓)
$\lambda_{\mathrm{CE}}=1,\;\lambda_{\mathrm{gIoU}}=1$	0.312	0.583	0.412
$\lambda_{\mathrm{CE}}=1.2,\;\lambda_{\mathrm{gIoU}}=1$	0.305	0.579	0.427
$\lambda_{\mathrm{CE}}=0.8,\;\lambda_{\mathrm{gIoU}}=1.2$	0.319	0.587	0.381
$\lambda_{\mathrm{CE}}=1,\;\lambda_{\mathrm{gIoU}}=1.2$	**0.324**	**0.589**	**0.373**

## Data Availability

The data are contained within the article.
